# Exploring the Crosstalk between Inflammation and Epithelial-Mesenchymal Transition in Cancer

**DOI:** 10.1155/2021/9918379

**Published:** 2021-06-14

**Authors:** Indranil Chattopadhyay, Rangarao Ambati, Rohit Gundamaraju

**Affiliations:** ^1^Department of Life Sciences, Central University of Tamil Nadu, Thiruvarur 610005, India; ^2^Department of Biotechnology, Vignan's Foundation for Science, Technology and Research Deemed to Be University, Vadlamudi, Di-522 213 Guntur, Andhra Pradesh, India; ^3^ER Stress and Mucosal Immunology Lab, School of Health Sciences, University of Tasmania, Launceston, Tasmania, Australia

## Abstract

Tumor cells undergo invasion and metastasis through epithelial-to-mesenchymal cell transition (EMT) by activation of alterations in extracellular matrix (ECM) protein-encoding genes, enzymes responsible for the breakdown of ECM, and activation of genes that drive the transformation of the epithelial cell to the mesenchymal type. Inflammatory cytokines such as TGF*β*, TNF*α*, IL-1, IL-6, and IL-8 activate transcription factors such as Smads, NF-*κ*B, STAT3, Snail, Twist, and Zeb that drive EMT. EMT drives primary tumors to metastasize in different parts of the body. T and B cells, dendritic cells (DCs), and tumor-associated macrophages (TAMs) which are present in the tumor microenvironment induce EMT. The current review elucidates the interaction between EMT tumor cells and immune cells under the microenvironment. Such complex interactions provide a better understanding of tumor angiogenesis and metastasis and in defining the aggressiveness of the primary tumors. Anti-inflammatory molecules in this context may open new therapeutic options for the better treatment of tumor progression. Targeting EMT and the related mechanisms by utilizing natural compounds may be an important and safe therapeutic alternative in the treatment of tumor growth.

## 1. Introduction

**Figure 1:**
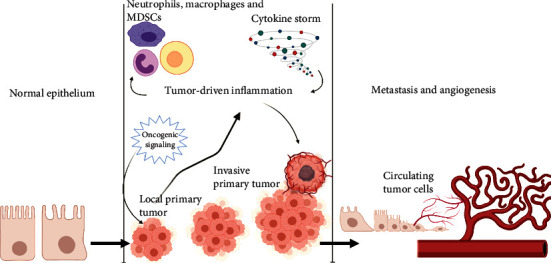
Crosstalk between inflammation and EMT in tumor progression. Proinflammatory cytokine molecules which are released by tumor cells drive epithelial-mesenchymal transition (EMT), invasion, and metastasis.

**Figure 2:**
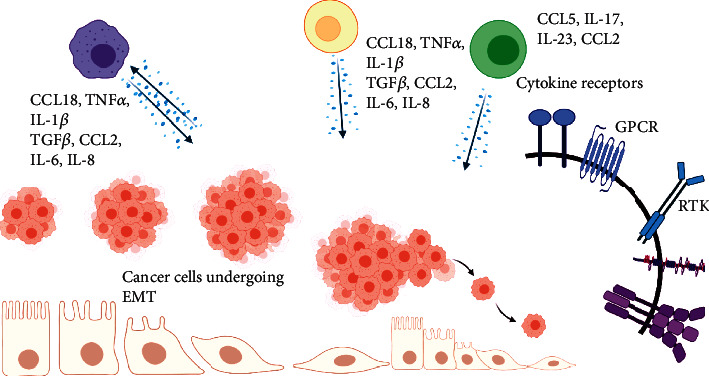
Schematic representation of inflammatory molecules released by immune cells that drive EMT. RTK: receptor tyrosine kinase; GPCR: G-protein-coupled receptor.

**Figure 3:**
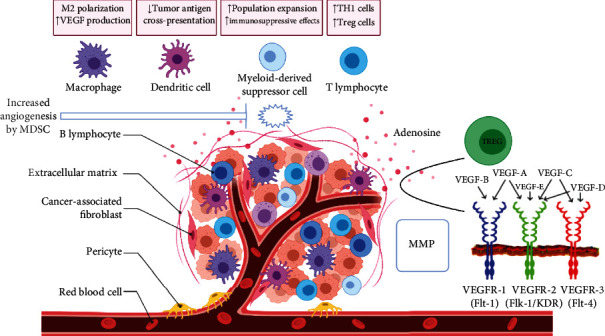
Role of tumor-associated macrophages (TAMs), myeloid cell-derived suppressor cells (MDSCs), cancer-associated fibroblasts (CAFs), and dendritic cells (DCs) in tumor microenvironment that induce angiogenesis through secretion of vascular endothelial growth factor (VEGF) and MMPs.

Genomic alterations including mutation and inactivation of tumor suppressor genes, cell proliferation, immune evasion, inflammation, invasion, angiogenesis, and metastasis are the hallmarks of cancer. Tumor cells undergo invasion and metastasis through epithelial-to-mesenchymal cell transition (EMT) by activation of alterations of extracellular matrix (ECM) protein-encoding genes, enzymes responsible for the breakdown of ECM, and activation of genes that drive the transformation of the epithelial cell to the mesenchymal type [1]. Inflammatory cytokines such as TGF*β*, TNF*α*, IL-1, IL-6, and IL-8 activate transcription factors such as Smads, NF-*κ*B, STAT3, Snail, Twist, and Zeb that drive EMT. The most important hallmark of EMT is the loss of E-cadherin expression and overexpression of mesenchymal cell proteins such as fibronectin, N-cadherin, vimentin, and matrix metalloproteinases (MMPs). EMT drives primary tumors to metastasize in different parts of the body (Figures 1 and 2 ) [2]. Inflammation contributes significantly to the tumor cell metastasis. T and B cells, dendritic cells (DCs), and tumor-associated macrophages (TAMs) which are present in the tumor microenvironment induce EMT. Vascular endothelial growth factor-A (VEGF-A), VEGF-C, and VEGF-D which are secreted by TAMs drive angiogenesis [3]. Fibroblasts, myofibroblasts, granulocytes, macrophages, myeloid cell-derived suppressor cells (MDSCs), mesenchymal stem cells, and lymphocytes which are present in the tumor microenvironments are responsible for the secretion of EMT-inducing signaling molecules [4]. Cancer-associated fibroblasts (CAFs) are responsible for the secretion of matrix-degrading enzymes, and growth factors such as FGF induce EMT (Figure 3) [5]. Here, we focus on the role of inflammatory proteins in EMT which drives invasion and metastasis of tumor cells. Developments of novel therapeutic targets against EMT are considered as major challenges in cancer treatment.

## 2. Role of Epithelial-Mesenchymal Transition (EMT) in Cancer

EMT is defined as a cellular process in which epithelial cells are converted to mesenchymal cells through alterations such as loss of contact inhibition and transformations of keratin to vimentin-type intermediate filaments [6]. EMT is responsible for the invasion and migration of tumor cells [7]. Transcription factors, such as Snail, Slug, Twist, and Zeb proteins, induce EMT through inactivation of cell junction proteins such as E-cadherin, CAR, claudins, and occludin [8]. During EMT, mesenchymal proteins such as vimentin, fibronectin, N-cadherin, and integrins are overexpressed whereas expression of cytokeratins is reduced [9]. *β*-Catenin, AP-1, and SP1 induced EMT through activation of mesenchymal proteins via TGF*β*1-Smads complex [10]. Snail induces downregulation of expression of metastasis suppressor genes such as Raf kinase inhibitor protein (RKIP) which prevents the MAPK and NF-*κ*B pathways as well as the function of tumor suppressor gene PTEN [11].

Tumor-associated macrophages (TAMs) are responsible for the secretion of inflammatory cytokines such as tumor necrosis factor alpha (TNF*α*) which induces EMT via activation of p38 MAPK [12]. NF-*κ*B regulates TNF*α*-mediated EMT in breast cancer cells. It also regulates EMT in pancreatic and colon cancer through overexpression of transcription factors such as Snail1 and LEF1 [13]. Matrix metalloproteinases, cathepsins, and urokinase (u-Pa) secreted by tumor-infiltrating immune cells induce EMT in the tumor microenvironment [14]. Myeloid-derived suppressor cells (MDSCs) secrete MMP9 which drive tumor cell invasion and metastasis [15]. MDSCs induce EMT of tumor cells through secretion of TGF*β* and EGF [16]. Lipopolysaccharide (LPS) of Gram-negative bacteria binds with Toll-like receptor 4 (TLR4) and initiates downregulation of E-cadherin whereas it upregulates mesenchymal markers such as S100A and *α*-SMA as well as TGF*β*1 which drive EMT. Flagellin and muramyl dipeptides (MDP) of bacteria bind with the TLR5 receptor which induces EMT through the production of TGF*β* [17].

## 3. Role of Inflammation in Tumorigenesis

Macrophages (Mfs) which are mononuclear cells are primarily localized in intestinal lamina propria. Type I macrophages (M1) which are mainly localized in chronic inflammatory sites and primary tumors are responsible for the secretion of proinflammatory cytokines such as tumor necrosis factor *α* (TNF*α*), and interleukin- (IL-) 12. Type II macrophages (M2) are responsible for the generation of inflammatory cytokines including IL-4, IL-13, and IL-10 which drive angiogenesis. Tumor-associated macrophages (TAMs) mainly belong to the M2 phenotype. IL-23 which is secreted by macrophages induces the production of IL-17 that drives the synthesis of IL-1, IL-6, IL-8, CXC ligand 1, and TNF*α* from epithelial, endothelial, and stromal cells [18]. Inflammatory cytokines (IL-1*β*, IL-6, and TNF*α*) and growth factors (HGF, EGF, TGF, and PDGF) which are secreted by TAMs are involved in the angiogenesis, immunosuppression, and breakdown of the extracellular matrix [19].

Activated M1 macrophages are responsible for the induction of Th1 responses through secretion of inflammatory cytokines such as IL-1, IL-6, IL-12, and TNF. M2 macrophages are responsible for the induction of Th2 responses through the secretion of anti-inflammatory cytokines [20]. Elevated levels of circulating MDSCs and IL-6 and IL-8 in serum have been reported in prostate cancer patients with poor prognosis [21]. IL-8 which induces EMT show higher levels in serum of cancer patients and hence may be considered to be a biomarker for poor clinical outcome in cancer patients [22]. Overexpression of IL-6 has been observed in breast, colon, non-small cell lung, pancreatic, prostate, and ovarian cancer. IL-6 binds with the IL-6 receptor which activates signal transducer and activator of transcription 3 (STAT3) pathways that drive tumor cell proliferation, EMT, migration, invasion, and metastasis [23]. Tumor-associated macrophages (TAMs) and CC-chemokine receptor 1 (CCR1)^+^ immature myeloid cells are responsible for the tumor invasion through IL-4-dependent secretion of matrix-degrading enzymes such as MMPs, cathepsins, and heparanase. CC- and CXC chemokine receptors such as CCR1, CCR4, CCR7, CCR9, CCR10, CXCR1–5, and CXCR7 exhibit overexpression in a tumor cell response to proinflammatory cytokines. These are involved in the metastasis of breast and colon cancer. CCL2, CXCL5, and CXCL12 are responsible for the recruitment of MDSCs at tumors which show immunosuppressive functions through the production of IL-10, TGF*β*, and arginase [24].

## 4. Role of Inflammatory Proteins in Influencing EMT of Cancer Cells

Inflammatory proteins are considered to be a potent inducer of EMT (Table 1). Activated human T cells are involved in the synthesis of IL-6, TNF*α*, and TGF*β* which induce the expression of mesenchymal proteins such as fibronectin, vimentin, and Zeb1 in inflammatory breast cancer cells [25]. Immune-stimulatory molecules such as CD80, CD86, and CD127 induced EMT in lung cancer with significant overexpression of CD4+ Foxp3+ regulatory T cells [26]. Tumor cell undergoes epithelial to mesenchymal transition (EMT) through secretion of IL-6, IL-8, GRO, GM-CSF, VEGF, and angiogenin [27]. Epidermal growth factor (EGF) induces EMT in the triple-negative breast cancer cell (TNBC) line through secretion of IL-6 and IL-8 [28]. EMT induces the development of resistance against anticancer therapy. Lung cancer cells developed resistance against EGFR tyrosine kinase inhibitors such as gefitinib and erlotinib due to EMT that drives overexpression of IL-6 and IL-8 [29]. TGF*β*1 induces overexpression of IL-6 in TAMs and T cells which are involved in the poor survival of breast cancer [30]. The NF-*κ*B pathway can be regulated by bacterial lipopolysaccharide (LPS), proinflammatory cytokines such as TNF*α* and IL-1, and DNA damage-inducing agents. I*κ*B kinase induces the phosphorylation of I*κ*Bs which drive the translocation of NF-*κ*B into the nucleus from the cytoplasm and induce the transcription of proinflammatory cytokine genes such as TNF*α*, IL-1, and IL-6. TNF*α* induces the development of tumors through reactive oxygen species- (ROS-) mediated DNA damage. NF-*κ*B triggers the activation of antiapoptosis mechanism through activation of BCL-2 and GADD45*β*. This generates invasion, metastasis, and angiogenesis of tumor through activation of VEGF, COX-2, MMP-9, and IL-8. The NF-*κ*B pathway may be considered as a connecting link between inflammation and tumorigenesis through activation of antiapoptotic genes, angiogenesis factors, and proinflammatory cytokines. TNF*α* induces colitis-associated CRC through the NF-*κ*B pathway. Infliximab may be considered as a therapeutic target for TNF*α* in colitis-associated CRC [31]. Overexpression of COX-2 has been reported in colorectal cancer. COX-2 induces inflammation-driven colorectal cancer through overexpression of antiapoptotic genes such as BCL-2 and MMPs. PPAR*δ* activates the expression of COX-2 in colonic cancer cells. COX-2 also activates PGE2 that articulates migration and invasion of the colonic epithelium through overexpression of proangiogenic chemokine CXCL1. It also induces macrophages to secrete proinflammatory cytokines that modulates colitis-associated colorectal cancer [31]. IL-17 drives angiogenesis in CRC through overexpression of VEGF. IL-17-producing helper T cells (Th17 cells) induce intestinal inflammation through the secretion of IL-21 and IL-22. IL-22 induces metastasis of colitis-associated CRC through activation of STAT3 and antiapoptotic proteins such as BCL-XL [32]. GMCSF-activated macrophages enhance EMT in breast cancer cells through CCL18 [33]. Activated neutrophils and eosinophils with overexpressed CD66b have been reported in lung adenocarcinoma. Expression of E-cadherin was negatively correlated with the expression of CD66b [34].

### 4.1. Transforming Growth Factor *β* (TGF*β*)

Cancer-associated fibroblasts (CAFs), leukocytes, endothelial cells, and immune-infiltrating cells (macrophages) are responsible for the secretion of anti-inflammatory cytokine TGF*β* [35]. Tumor-associated macrophages (TAM), myeloid-derived suppressor cells (MDSCs), and regulatory T cells (Treg) which are present in the tumor microenvironment are responsible for the production of TGF*β*1 which orchestrates tumor metastasis and EMT-permissive tumor microenvironment [36]. TGF*β* induces chemotaxis of eosinophils, macrophages, and mast cells and prevents antitumor immune response by blocking antigen-presenting functions of DCs, cytotoxic functions of CD8+ T cells, and activation of inflammatory CD4 T cells such asTh17 or Th9 [37].

TGF*β* functions as a tumor suppressor by blocking cell proliferation through overexpression of cyclin kinase inhibitors (CKIs) and inducing programmed cell death in an early stage of tumor. It also induces the expression of CDKN2B through activation of binding of the Smad2/3–Smad4–Foxo complex to the promoter that inhibits DNA methylation by recruiting the DNA excision repair complex which includes DNA glycosylases, thymine DNA glycosylase (TDG), and methyl-CpG-binding domain-4 (MBD4) [38]. TGF*β* induces tumor metastasis through evasion of immune cell function and activation of angiogenesis and EMT [39]. TGF*β* induces inactivation of CDH1 in human Panc1 cells through activation of Snail1 and Snail2 [40]. It additionally activates EMT through enablement of Ras/Raf/MAPK, phosphatidylinsitol-3 (PI3) kinase/Akt, NF-*κ*B signaling, and the Rho/Rac1 and Cdc42 GTPases [41]. TGF*β* induces EMT of mammary epithelial cells through overexpression of SIRT1 deacetylase (NAD-dependent deacetylase sirtuin-1) that organises deacetylation of histone and inactivation of a promoter of the miR-200 gene [42]. Transforming growth factor *β* (TGF*β*) modulates EMT through the SMAD-dependent pathway via activation of Slug and Snail in malignant mammary epithelial cells [43].

### 4.2. Tumor Necrosis Factor Alpha (TNF*α*)

The tumor necrosis factor alpha (TNF*α*) which is an inflammatory cytokine binds with TNF receptors such as TNFR1 (CD120a) and TNFR2 (or CD120b). It activates caspase-mediated apoptosis, MAPK (ERK, JNK, and p38*α*), and canonical NF-*κ*B signaling pathways. It incites inflammation and metastasis through NF-*κ*B-mediated activation of IL-6, IL-8, IL-18, chemokines, inducible nitric oxide synthase (iNOS), cyclooxygenase-2 (COX-2), and 5-lipoxygenase (5-LOX). TNF*α* exhibits antitumor effects through activation of programmed cell death [44] and migration and invasion of breast cancer cells through activation NF-*κ*B-dependent EMT-inducing transcription factors (EMT-TFs) such as Twist1, Snail, Slug, and Zeb1/2 that drive inactivation of E-cadherin. TNF*α* in addition activates TGF*β*-mediated EMT and EMT in colon cancer and lung epithelial cancer cell lines through activation of miR-21, miR-31, and miR-23a [2]. TNF*α* further induces inflammation through NF-*κ*B-dependent activation of miR-155 [45]. Suppression of EZH2 lysine methylase induces NF-*κ*B-dependent TNF*α*-mediated inflammatory responses through activation of TRAF2/5 [46]. It induces EMT in colon cancer through overexpression of NF-*κ*B-dependent miR-105 expression [47]. TNF*α* along with TGF*β* triggers EMT in colon cancer cells through activation of Snail1, claudin-1, and the NOD-like receptor family, pyrin domain containing 3 (NLRP3) [48], and activates EMT in renal cell carcinoma through overexpression of chemokine receptors such as CXCR2 and CXCR3 [49].

### 4.3. Interleukin-1*β* (IL-1*β*)

Transcription factors such as AP-1 and NF-*κ*B induce expression of IL-1*β* in immune cells. Expression of IL-1*α* is also induced by Sp1, AP-1, and NF-*κ*B. IL-1 induces secretion of inflammatory cytokines and chemokines after binding with IL-1R1 which has immunoglobulin (Ig) domains and Toll-like/IL-1R (TIR) domain through the canonical NF-*κ*B signaling pathway. IL-1*α*, IL-1*β*, and IL-1R which are commonly expressed by tumor-infiltrating immune effector cells and tumor stromal cells are responsible for shaping the tumor microenvironment. Inflammatory mediators induce proliferation and survival of tumor cells [50]. It additionally induces EMT through overexpression of stemness markers Bmi1 and Nestin which maintain self-renewal of cancer stem cells (CSCs) [51]. In a pancreatic ductal carcinoma (PDAC) model, mutation of K-Ras enhances the expression of IL-1 which drives progression and invasion of the tumor through constitutive activation of NF-*κ*B [52]. IL-1*β* along with TGF*β*3 increases invasiveness of lung epithelial cancer cells through inducing secretion of MMPs [53]. IL-1*β* induces tamoxifen resistance in the breast cancer cell model via prompting of Twist1 that propels methylation in the promoter region of the ESR1 gene which in turn reduces the expression of Er*α* [54]. IL-1*β* induces EMT through overexpression of Zeb1 and reduced expression of CDH1 via NF-*κ*B [55]. IL-1*β* induces EMT, invasion, and chemotherapeutic drug resistance in a breast cancer cell model through activation of cIAP2, c-Myc, CCDN1, MMP2, and Snail1 genes [56]. IL-1*β* induces expression of inflammatory cytokine genes IL-6 and IL-8 through CpG demethylation at the promoter sites of the IL-6 and IL-8 in human colon cancer epithelial Caco2 cells [57]. It further enhances the survival of gastric tumor cells through upregulation of NF-*κ*B-dependent miR-425 which targets tumor suppressor gene PTEN [58]. It activates inflammation in gastric tumors with TNF*α* through reduced expression of miR-7 [59]. IL-1*β* produces inflammation in NSCLC cells through downregulation of miR-101 expression via the Cox2–HIF1*α* pathway [60] and supports proliferation of colon tumors through the miR-181a/PTEN axis [61]. It also induces the growth of osteosarcoma through reduced expression of miR-506 via the JAG1-mediated Notch signaling pathway [62]. In oral cancer, IL-1*β* enhances EMT in oral squamous cell carcinoma and dysplastic oral keratinocytes through the production of proinflammatory cytokines such as IL-6, IL-8, and GRO*α* [63].

### 4.4. Interleukin-6 (IL-6)

Tumor cells and reactive stroma are responsible for the synthesis of IL-6 which induces inflammation [64] via Ras/Raf/MAPK, PI3K, or Src/YAP pathways through JAK [65]. NF-*κ*B, STAT3, C/EBP, CREB, and AP-1 trigger expression of IL-6 which drives tumorigenesis via the Ras/Raf/MEK and PI3Kpathways [2]. IL-6 activates CpG island methylation in promoter regions of the p53 tumor suppressor gene which articulates tumor cells to bypass cell cycle checkpoints [66] and induces chronic inflammation in oral cancer through CpG promoter methylation of tumor suppressor genes such as CHFR, GATA5, and PAX6 [67]. IL-6 aids in EMT via JAK-STAT3 or NF-*κ*B pathways by activating EMT-TFs such as Snail, Slug, Twist, and Zeb1 which in turn reduces expression of CDH1 that drives migration and invasion of the tumor. IL-6 induces STAT3-driven EMT, invasion, and metastasis in colorectal cancer through downregulation of miR-34a which prevents EMT through regulation of Snail1 [2]. It also adds to the aggressiveness of glioblastoma through hypermethylation of a Sp1-binding site in the miR142-3p gene [68]. In aggressive metastatic ovarian carcinoma, tumor-associated macrophages (TAMs) are involved in the secretion of IL-6 which induces EMT via activation of STAT3. STAT3 induces the development of chemoresistance in ovarian cancer [69]. IL-6 binds with IL-6R to form an IL-6/IL-6R complex which activates the JAK/STAT, Ras/ERK, and PI3K/Akt pathways. The IL-6/STAT3 pathway supports proliferation of premalignant intestinal epithelial cells (IEC) which drive in colitis-associated cancer [70]. Tropomyosin receptor kinase C activates IL-6 which activates EMT through upregulation of Twist1 [71]. IL-6 triggers EMT in lung adenocarcinoma through activation of STAT3/Snail1 [72].

### 4.5. Interleukin-8 (IL-8)

IL-8 activates the Ras/Raf/MAPK, PI3K, or JAK/STAT pathways through binding with G-protein-coupled receptors such as CXCR1/2. Erlotinib which targets the epidermal growth factor receptor (EGFR) tyrosine kinase sets off secretion of IL-8 that coordinates EMT through the p38 MAPK kinase pathway [48]. Snail1 also plays a role in the activation of EMT in colon cancer cells through transcriptional activation of the IL-8 gene by binding with E-box motifs which are present in the promoter region in the IL-8 gene [73]. IL-8 integrates EMT in breast, colon, thyroid, and nasopharyngeal cancer through activation of a Slug-Akt signaling pathway [23]. Zonula occludens-1 (ZO-1) which is a tight junction protein-1 induces the NF-*κ*B dependent synthesis of IL-8 in breast and lung cancer cell lines [74]. IL-8 in other scenarios activates EMT in thyroid cancer cells and hepatocellular carcinoma via overexpression of the AKT/Slug and JAK2/STAT3/Snail1 pathways, respectively [75, 76], and in nasopharyngeal carcinoma through epigenetic silencing of E-cadherin [77].

### 4.6. Chemokines

CCL2 (or monocyte chemotactic protein 1 (MCP1)) displays overexpression in TAM [78] and triggers EMT in lung cancer with IL-6 through activation of Twist/STAT3 [79]. The ER*β*/CCL2/CCR2 axis also initiates EMT in bladder cancer [80]. Proinflammatory chemokine CCL5 triggers key mechanisms such as EMT, angiogenesis, and metastasis [81] and binds with CCR1, CCR3, and CCR5 which are present on the surface of myeloid cells, T cells, and tumor cells [48]. It is produced by CD133-positive stem cells in ovarian cancer and sets up EMT and metastasis of CD133-negative stem cells through activation of NF-*κ*B in ovarian cancer [82]. It additionally triggers EMT in triple-negative breast cancer cells [83] and liver metastasis in colon cancer patients [84]. CCL18 secreted by M2 macrophages induces EMT in pancreatic cancer through activation of Snail1 [86]. It is secreted by TAMs and triggers EMT and invasion of breast cancer cell lines through overexpression of vimentin and downregulation of E-cadherin [33]. CCL18 which endorses EMT in ovarian cancer cells showed a positive correlation with metastasis in ovarian cancer patients [87] and further triggered EMT in the colon and hepatocellular carcinoma. It binds with CCR6 receptors which showed higher abundance in aggressive tumors [88, 89]. CCL20 was overexpressed in hepatocellular carcinomas with poor prognosis [89]. It triggers EMT and invasion through overexpression of N-cadherin and MMP9 via the NF-*κ*B-mediated pathway [90]. TGF*β*1 activates EMT of CCL21-positive breast cancer cells [91].

## 5. Role of EMT and Inflammation Controlling Cancer Stem Cell (CSC) Properties

EMT is considered to be a key player to maintain the stemness properties of tumor cells [92]. Proinflammatory cytokines are involved in the regulation of reprogramming and renewal of CSC through STAT3. IL-1, TNF*α*, and IL-6 induce metastasis via activation of NF-*κ*B and STAT3 [6]. TNF*α* induces inflammation in gastric cancer through activation of *β*-catenin [93], and NF-*κ*B triggers the transcriptional activation of IL-6 in head and neck tumors [6]. It has been reported that TGF*β* is transcriptionally active in CD44(+) breast cancer stem cells [94]. TWIST1 induces the differentiation of ovarian cancer stem cells through overexpression of hsa-miR-199a/hsa-miR-214 via the NF-*κ*B and PTEN/AKT pathways that drive EMT-mediated inflammation [95].

## 6. Therapeutic Potential of Natural and Other Compounds in Regulation of EMT

Natural compounds have successfully exhibited anti-inflammatory and anti-EMT activities (Table 2) [96]. Genistein blocks TGF*β*-dependent invasion and metastasis of the pancreatic cancer cell line (Panc-1) via Smad4-dependent and p38 MAPK pathways [97]. It further transforms EMT to MET in a hepatocellular carcinoma cell line (HepG2) through suppression of the nuclear factor of activated T cells 1 (NFAT1) [98]. Genistein prevented endocrine disruptor-induced EMT in ovarian cancer cells (BG-1) through suppression of the TGF*β* pathway [99]. Resveratrol on the other hand displayed inhibitory activity against invasion and metastasis of gastric cancer through suppression of EMT [100]. It additionally prevented hypoxia-induced EMT in osteosarcoma cells through suppression of the HIF-1*α* protein [101]. It impeded LPS-dependent EMT in prostate cancer cell lines (PC-3 and LNCaP) through blocking of the Hh signaling pathway [102] and impeded the EMT process in colorectal cancer cells through overexpression of E-cadherin and suppression of vimentin by blocking the TGF*β*1/Smads signaling pathway [103]. Resveratrol exhibited reprogramming of EMT into MET in pancreatic cancer via suppression of AKT signaling pathways [104] and prevented EGF-mediated activation of EMT in the ER-positive breast cancer cell line (MCF-7) through suppression of the EGF-activated Erk pathway [105]. Resveratrol presented inhibitory action against TGF*β*1-induced EMT in lung cancer cells [106].

Kaempferol is another successful natural product which offered inhibitory action against EMT through suppression of mesenchymal protein expression in non-small cell lung cancer [107]. The application of phytoestrogens is considered to be an important treatment of cancer by blocking the EMT process [108]. Arctigenin (ARC) prevented TGF*β*-induced EMT in lung cancer cells [109]. Baicalin and baicalein suppressed the TGF*β*1-dependent EMT process in mammary epithelial cells through blocking of the expression of the Slug protein and NF-*κ*B signaling pathway [110]. Berberine suppressed EMT in tumor cells through overexpression of E-cadherin and suppression of N-cadherin, fibronectin, vimentin, Snail, Slug, and zinc finger E-box binding homeobox 1 (Zeb1) expression [111]. Celastrol reduced the expression of proinflammatory cytokines (IL-1*β*, IL-6, and TNF*α*), cyclooxygenase 2 (COX-2), N-cadherin, Vimentin, and Snail. It induces the expression of E-cadherin [112]. Epicatechin-3-gallate (ECG) is involved in the TGF*β*1-dependent EMT through overexpression of E-cadherin and inhibition of mesenchymal proteins in lung cancer cells [113]. Gedunin also revealed inhibitory activity against the EMT process through declination of mesenchymal proteins such as Slug, Snail, N-Cadherin, Vimentin, and Zeb as well as upregulation of E-cadherin [114]. Plumbagin (PLB) induces overexpression of E-cadherin and reduces expression of Snail, Slug, TCF-8/Zeb1, *β*-catenin, and vimentin which drive reprogramming of the EMT process [115]. Cardamonin prevents EMT in triple-negative breast cancer cells by blocking the Wnt signaling pathway. Similarly, curcumin inhibits TNF*α*-dependent EMT in tumor cells. Luteolin also inhibits TGF-1-dependent EMT in lung cancer cells by blocking the PI3K/AKT/NF-*κ*B/Snail pathway and also inhibits IL-6-dependent EMT in pancreatic cancer through the prevention of STAT3 signaling. It further showed anti-EMT activity in paclitaxel-resistant ovarian cancer cells. Nimbolide also showed anti-EMT activity [96]. Monoclonal antibodies (mABs) and small molecule inhibitors such as Fresolimumab (GC-1008), Erlotinib, Gefitinib, Lapatinib, Sorafenib, Vandetanib, Niclosamide, Onartuzumab, Everolimus, and Temsirolimus are employed for the regulation of EMT in several tumors [116].

Nonsteroidal anti-inflammatory (NSAIDs) drugs such as apricoxib, celecoxib, etodolac, and sulindac are involved in the suppression of TGF*β*1/EGF-induced EMT and inflammation via blocking the expression of Slug, Snail1, vimentin, and Zeb1 proteins as well as overexpression of E-cadherin. Dexamethasone offered inhibitory activity against hypoxia and TGF*β*1-induced EMT of tumor cells by blocking the expression of transcription factors such as Snail1, Slug, and Twist. Calcitriol blocks LPS-induced IL-6 and TNF*α*-dependent EMT by inducing the overexpression of E-cadherin. Simvastatin prevents TGF*β*1-induced EMT in lung and prostate cancer cells by arresting phosphorylation of Smad2 and Smad3 proteins [117]. Natural-derived STAT3/5 inhibitors such as Cryptotanshinone, Capsaicin, Cucurbitacin I, Celastrol, and Sulforaphane are utilized to prevent inflammation by inhibiting the action of upstream tyrosine kinases which are involved in the phosphorylation of STAT3/5 [118]. Combinatorial approaches such as EMT inhibitors along with immunotherapy such as anti-PD1/PD-L1- and CTLA4-associated treatments are promising therapeutic strategies [119] especially drugs such as Infliximab which is chimeric anti-TNF antibody that prevents TNF-dependent inflammation through blocking the interaction between TNF and its receptor [120].

## 7. Conclusion and Future Perspective

Proinflammatory cytokines (TNF*α* and IL-6), chemokines (CCL2, CCL5, and CXCL12), and CXCL8 receptors (CXCR1 and CXCR2) are involved in the regulation of stemness and EMT property in breast cancer cells. CD90 which is expressed by tumor cells induces interaction between the tumor cells and macrophages. This orchestrates the overexpression of IL-6, CXCL8, and granulocyte macrophage that constitute the formation of tumor spheres. Proinflammatory cytokines such as TNF*α*, IL-6, and inflammatory chemokines such as CXCL8 and CXCL1 contribute significantly in stemness, EMT, and resistance to chemotherapy. Overexpression of CXCR1/CXCR2 and CXCL8 in tumor indicates poor prognosis of the diseases [121]. Mesenchymal stem cells (MSCs) within the tumor microenvironment induce EMT. The differentiation of MSCs into cancer-associated fibroblasts (CAFs) triggers EMT through the CXCL12/CXCR4 axis [122].

EMT influences cytokine storm in the tumor cells. Inflammation, on the other hand, not only contributes in cancer initiation but also is involved in a series of events like cell death evasion, survival, and EMT and eventually making chemoresistance as the fate. Inflammatory cells of the tumor and its microenvironments trigger EMT. Inflammatory cytokines such as TGF*β*, TNF*α*, IL-1, and IL-6 may induce activation of Snail, Twist, and Zeb proteins that drive EMT via activation of Smads, NF-*κ*B, and STAT3. A better apprehension of EMT and inflammation crosstalk may be considered as a better understanding of tumor angiogenesis and metastasis and pave pathways for novel therapeutic options for the better treatment of tumors in diverse cancers since targeting inflammation and EMT is pivotal to combat chemoresistance in cancers. Understanding the anti-inflammatory and anti-EMT activities of natural compounds may provide a new avenue to suppress the growth of tumors.

## Figures and Tables

**Table 1:** Role of inflammatory proteins in regulation of EMT in tumor cells.

Inflammatory factors and its sources	Role of EMT in tumor cells	References
TNF*α* (secreted by macrophages)	It induces EMT in breast cancer cells through activation of NF-*κ*B-dependent transcription factors (EMT-TFs) such as Twist1, Snail, Slug, and Zeb1/2 that drive inactivation of E-cadherin	[2]
	It activates EMT in renal cell carcinoma through overexpression of chemokine receptors such as CXCR2 and CXCR3	[49]
TGF*β* (secreted by cancer-associated fibroblasts, leukocytes, endothelial cells, and immune-infiltrating cells)	It induces inactivation of CDH1 in human Panc1 cells through activation of Snail1 and Snail2	[40]
	It induces EMT of mammary epithelial cells through overexpression of SIRT1 deacetylase (NAD-dependent deacetylase sirtuin-1) that drives deacetylation of histone and inactivation of a promoter of the miR-200 gene	[42]
IL-1*β* (secreted by tumor-infiltrating immune effector cells and tumor stromal cells)	It also induces EMT through overexpression of stemness markers Bmi1 and Nestin which maintain self-renewal of cancer stem cells (CSCs)	[51]
	It induces tamoxifen resistance in the breast cancer cell model through activation of Twist1 that drives methylation in the promoter region of ESR1 gene which in turn reduces the expression of ER*α*	[56]
	It enhances EMT in oral squamous cell carcinoma and dysplastic oral keratinocytes through the production of proinflammatory cytokines such as IL-6, IL-8, and GRO*α*	[63]
IL-6 (secreted by T cells, macrophages, and tumor cells)	It induces EMT via JAK-STAT3 or NF-kB pathways via activation of EMT-TFs such as Snail, Slug, Twist, and Zeb1 which reduces the expression of CDH1 that drive migration and invasion of the tumor. It induces STAT3-driven EMT, invasion, and metastasis in colorectal cancer through downregulation of miR-34a which prevents EMT through regulation of Snail1	[2]
	It induces EMT via activation of STAT3 which induces the development of chemoresistance in ovarian cancer	[69]
	It triggers EMT in lung adenocarcinoma through activation of STAT3/Snail1	[72]
IL-8 (secreted by T cells, macrophages, and tumor cells)	It induces EMT in breast, colon, thyroid, and nasopharyngeal cancer through activation of a Slug-Akt signaling pathway	[23]
	It induces EMT in thyroid cancer cells and hepatocellular carcinoma through overexpression of the AKT/Slug and JAK2/STAT3/Snail1 pathways, respectively	[75, 76]
	It triggers EMT in nasopharyngeal carcinoma through epigenetic silencing of E-cadherin	[77]
CCL2 (secreted by monocytes, macrophages, and dendritic cells)	It triggers EMT in lung cancer with IL-6 through activation of Twist/STAT3	[79]
CCL5 (secreted by cancer stem cells)	It triggers EMT and metastasis of CD133-negative stem cells through activation of NF-*κ*B in ovarian cancer	[86]
	It triggers EMT in triple-negative breast cancer cells	[83]
CCL18 (secreted by macrophages)	It induces EMT in pancreatic cancer through activation of Snail1	[86]
	It triggers EMT and invasion of breast cancer cell lines through overexpression of vimentin and downregulation of E-cadherin	[33]
CCL20 (secreted by lymphocytes)	It triggers EMT in the colon and hepatocellular carcinoma	[88, 89]
CCL21 (secreted by lymphocytes)	It triggers EMT and invasion through overexpression of N-cadherin and MMP9 via the NF-*κ*B-mediated pathway	[90]

**Table 2:** Natural compounds involved in modulation of epithelial-to-mesenchymal transition (EMT).

Natural compounds	Mode of action in blocking of EMT	References
Genistein	Suppress nuclear factor of activated T cells 1 (NFAT1) which induces MET in a hepatocellular carcinoma cell line (HepG2)	[98]
	Suppress the TGF*β* pathway that inhibits endocrine disruptor-induced EMT in ovarian cancer cells (BG-1)	[99]
Resveratrol	Suppress HIF-1*α* protein which inhibits hypoxia-induced EMT in osteosarcoma cells	[101]
	Inhibits the Hh signaling pathway which inhibits LPS-dependent EMT in prostate cancer cell lines (PC-3 and LNCaP)	[102]
	Induces overexpression of E-cadherin and suppression of vimentin by blocking the TGF*β*1/Smads signaling pathway in colorectal cancer cells	[103]
	Induce MET in pancreatic cancer via suppression of AKT signaling pathways	[104]
	Suppress the EGF-activated Erk pathway which prevents EGF-activated EMT in the ER-positive breast cancer cell line (MCF-7)	[105]
	Showed inhibitory action against TGF*β*1-induced EMT in lung cancer cells	[106]
Kaempferol	Suppress mesenchymal protein expression in non-small cell lung cancer	[107]
Arctigenin (ARC)	Prevented TGF*β*-induced EMT in lung cancer cells	[108]
Baicalin and baicalein	Block expression of Slug protein and NF-*κ*B signaling pathway that inhibit the TGF*β*1-dependent EMT process in mammary epithelial cells	[110]
Berberine	Induces overexpression of E-cadherin and suppression of N-cadherin, fibronectin, vimentin, Snail, Slug, and zinc finger E-box binding homeobox 1 (Zeb1) protein expression	[111]
Celastrol	Suppressed the expression of proinflammatory cytokines (IL-1*β*, IL-6, and TNF*α*), cyclooxygenase 2 (COX-2), N-cadherin, Vimentin, and Snail. It induces the expression of E-cadherin	[112]
Epicatechin-3-gallate (ECG)	Induces overexpression of E-cadherin and suppression of mesenchymal proteins which prevent TGF*β*1-dependent EMT in lung cancer cells	[113]
Gedunin	Downregulates expression of mesenchymal proteins such as Slug, Snail, N-cadherin, vimentin, and Zeb as well as upregulation of E-cadherin	[114]
Plumbagin	Induces overexpression of E-cadherin and reduces expression of Snail, Slug, TCF-8/Zeb1, *β*-catenin, and vimentin which drive reprogramming of the EMT process	[115]
Cardamonin	Blocks the Wnt signaling pathway which prevents EMT in triple-negative breast cancer cells	[96]
Luteolin	(i) Inhibits the PI3K/AKT/NF-kB/Snail pathway which prevents TGF-1-dependent EMT in lung cancer cells (ii) Prevents STAT3 signaling which inhibits IL-6-dependent EMT in pancreatic cancer (iii) Showed anti-EMT activity in paclitaxel-resistant ovarian cancer cells	[96]

## Data Availability

No data were used to support this study.
